# Insights into the mechanism of substrate specificity in a novel PL15_3 subfamily oligo-alginate lyase VBAly15A

**DOI:** 10.1128/aem.02351-24

**Published:** 2025-02-27

**Authors:** Yongqi Tang, Ziyan Song, Xiaodong Xu, Yingjie Li, Lushan Wang

**Affiliations:** 1 State Key Laboratory of Microbial Technology, Shandong University 520252 , Qingdao, China; 2 Qingdao Vland Biotech Company Group 641181 , Qingdao, China; Georgia Institute of Technology, Atlanta, Georgia, USA

**Keywords:** alginate, alginate lyase, catalytic mechanism, substrate specificity

## Abstract

Alginate is a major component of brown algae cell walls and can be degraded via β-elimination by alginate lyases. These enzymes are classified into polysaccharide lyases and oligo-alginate lyases (Oals), with Oals mainly represented by the PL15 and PL17 families. Unlike PL17 Oals, which are widely present in alginate-degrading microorganisms, PL15 enzymes are only identified in a limited number of microorganisms, and their biochemical characteristics remain poorly understood. In this research, a novel PL15 alginate lyase, VBAly15A, from the marine bacterium, *Vibrio* sp. B1Z05, was identified and characterized. It belongs to a new PL15_3 subfamily and exhibits high activity toward polyM substrates. VBAly15A is thermostable in medium temperatures, tolerant to alkaline up to 11.0, and polyM-specific Oal, and it can first degrade alginate polymers into disaccharides and subsequently catalyze disaccharides into monomers via an exolytic mode. Site-directed mutagenesis showed that Arg^114^, Tyr^470^, and Arg^110^ in the active groove are essential for the stable binding of the substrate. In addition, the amino acid His^226^ in VBAly15A, previously suggested to act as a catalytic base, is not essential for catalysis, whereas Tyr^280^, previously proposed to act as a catalytic acid, is required for enzyme activity. Structural bioinformatic and biochemical analyses revealed that His^226^ functions as a catalytic base, specifically abstracting protons from G-type substrates, while Tyr^280^ acts as both a catalytic acid and a base. This catalytic mechanism is likely conserved in PL15 family alginate lyases.

**IMPORTANCE:**

Alginate, as a renewable resource for sustainability, has great application prospects. In addition to polysaccharide lyases, Oals are critical for the full degradation of alginate, a key prerequisite for biorefinery. So far, most identified and well-characterized Oals belong to the PL17 family. However, the catalytic mechanism of PL15 Oals is limited, and even the catalytic base and acid are not fully elucidated. The significance of this study lies in discovering and characterizing a novel Oal VBAly15A that divides into a new PL15 subfamily, PL15_3. Not only are key amino acid residues involved in enzyme activity identified, but residues acting as the catalytic base and acid are also demonstrated. The distance of the catalytic residues His and Tyr to the C5 proton of the sugar ring determines the substrate specificity. Therefore, this work provides new insights into the mechanism of substrate specificity in alginate lyases.

## INTRODUCTION

Brown macroalgae are the most abundant seaweed worldwide, and they are mainly composed of alginate, mannitol, and glucan (in the form of cellulose and laminarin) (1, 2). Among these, alginate is the primary component of the cell wall of brown seaweed, and its content could reach up to more than 40% of the dry weight (3). Alginates consist of two conformational isomers, named β-D-mannuronic acid (M) and α-L-guluronic acid (G), which could be lined into three different types of alginate polymers: homopolymers polyG and polyM together with the heteropolymer polyMG (4). The ratios of G and M of brown algae are varied with species, tissue, season, or life cycle stage controlled by mannuronan C-5 epimerases (ManC5-Es) (5–8). In addition to seaweed, some microorganisms are also capable of synthesizing alginates, which are important for biofilm formation to defend against antibiotics (9–12). However, compared to those from brown algae, alginates originated from bacteria are acetylated on their O-2 and/or O-3 of mannuronate catalyzed by mannuronate acetylase (13, 14).

Alginate is considered a renewable resource and decomposed by alginate lyases through the β-elimination reaction. The CAZy database classifies alginate lyases into 16 families of polysaccharide lyases (PLs), namely, PL5, PL6, PL7, PL8, PL14, PL15, PL17, PL18, PL31, PL32, PL34, PL36, PL38, PL39, PL41, and PL44 (15, 16). These alginate lyases could be grouped as G-specific (EC 4.2.2.11), M-specific (EC 4.2.2.3), or bifunctional lyases (EC 4.2.2.-) based on their preferred substrates (17, 18). According to their catalysis mode, alginate lyases are divided into two groups: endo-type (4.2.2.-) and exo-type lyases (EC 4.2.2.26). The endo-type alginate lyases are mainly responsible for the extracellular conversion of alginates into alginate oligosaccharides (AOSs), whereas most exolytic enzymes are located in the periplasm or the cytoplasm and function as oligo-alginate lyases (Oals) to degrade AOSs into unsaturated monosaccharides, 4-deoxy-l-*erythro*-4-hexenopyranuronate (Δ). Therefore, not only endo-type alginate lyases but also Oals are required to achieve the full degradation of alginates. To this end, microorganisms usually contain a series of endo-type alginate lyases and Oals, and PL6 and PL7 family polymer lyases and PL17 family Oals are the most common lyases widely present in different bacteria (19, 20). However, an exception occurs in the marine bacterium *Falsirhodobacter* sp. alg1, which only relies on one copy of endolyase and one exo-type Oal to support the growth with alginate (21).

In general, alginate polymer lyases are responsible for the conversion of alginates into AOSs and exhibit great varieties in many aspects, including PL family, domain composition, action mode, product distribution, and protein structure, thereby achieving efficient degradation of alginate (22–25). However, Oals are mainly limited to the PL15 and PL17 families and contain similar component compositions: an alginate lyase domain at the N-terminus and a heparinase II/III-like domain at the C-terminal. Our previous study revealed that a pair of PL17 Oals is capable of fully digesting AOSs in alginate-degrading *Vibrio* species (submitted). During this process, one PL17 Oal mainly converts larger AOSs into disaccharides, while the other PL17 Oal is specific for the degradation of disaccharides. Their different roles in AOS metabolism likely result from one key loop, named Loop1, around the active site. Moreover, the size of Loop1 could serve as a critical factor in predicting the minimal substrates of different PL17 Oals. Different from the PL17 Oals that are widely observed in alginate-degrading microorganisms, the occurrence of the PL15 Oals is more accidental (19, 20). To date, only six Oals belonging to the PL15_1 subfamily have been identified and characterized, including A1-IV and A1-IV' from *Sphingomonas* sp. A1 (26, 27), Atu3025 from *Agrobacterium tumefaciens* strain C58 (28), OalA from *Vibrio splendidus* 12B01 (29), AlyFRB from *Falsirhodobacter* sp. alg1 (21), and AlyPB2 from *Photobacterium* sp. FC615 (30). Most of them are polyM-specific (15). Among these PL15 Oals, only the structure of Atu3025 was solved, which contains an (α/α)_6_ toroid and an anti-parallel β-sheet. Its residues His^311^ and Tyr^365^ in the active site are proposed to act as the catalytic base and the acid for the β-elimination reaction, respectively (31). However, the biochemical characteristics of the PL15 Oals are still largely unknown.

In this work, a novel PL15 Oal VBAly15A was identified from a marine alginate-degrading bacterium, *Vibrio* sp. B1Z05, which was isolated from an abalone gut and exhibits high efficiency in alginate degradation (32). Our bioinformatic data suggested that VBAly15A belongs to a new subfamily of PL15, PL15_3. Structural bioinformatic and biochemical analyses were performed to understand the catalytic mechanism of VBAly15A and its key amino acid residues involved in enzyme activity. In addition, the critical factor(s) related to the substrate preference were investigated using site-directed mutagenesis and molecular dynamic simulation. Our data revealed that VBAly15A is a polyM-specific exolytic Oal, and its catalytic acid Tyr^280^ may also function as a catalytic base. The distance between the catalytic residues His and Tyr in the active site and the C5 proton of the sugar ring at the +1 position is a key factor in determining substrate specificity in PL15 alginate lyases.

## RESULTS

### VBAly15A belongs to a new PL15 subfamily alginate lyase

**Fig 1:**
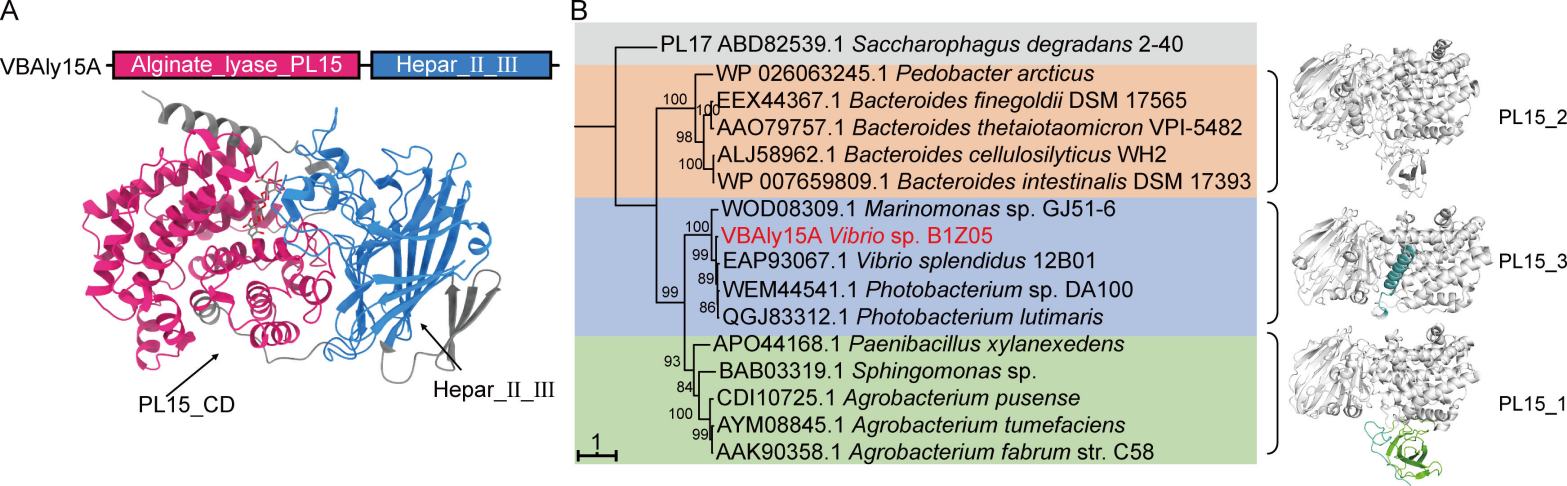
Sequence analysis of the alginate lyase VBAly15A from *Vibrio* sp. B1Z05. (**A**) Modular analysis and protein structure prediction with AlphaFold2. (**B**) Phylogenetic analysis of VBAly15A. The maximum likelihood estimation method was used to construct the phylogenetic tree on the Fast Tree, and 1,000 times of bootstrapping analysis was conducted. The right side of the phylogenetic tree shows the protein structures of each subfamily.

Our early study revealed that a pair of PL17 Oals is capable of fully digesting AOSs in alginate-degrading *Vibrio* species (submitted). However, PL15 Oal is not widely present in alginate-degrading microorganisms. Even microorganisms harbor PL15 Oals, and only a single PL15 member was usually observed. To understand the role of PL15 Oals in alginate saccharification, a PL15 Oal VBAly15A from *Vibrio* sp. B1Z05 (GenBank accession number: WP_023403303.1) (32) was investigated. As shown in Fig. 1A, VBAly15A has a similar domain organization with PL17 Oals consisting of an alginate lyase domain at the N-terminus (Asp^36^-Tyr^387^) and a heparinase II/III-like domain at the C-terminus (Ser^405^-Asn^661^). So far, two PL15 subfamily enzymes have been identified, one annotated as the PL15_2 heparinase and the other as the PL15_1 Oal. Phylogenetic analysis indicated that VBAly15A, OalA from *V. splendidus* 12B01 (29), and some Oals cluster in a single group (Fig. 1B), and sequence similarity analysis showed that VBAly15A shares low sequence similarities (57%) with the identified PL15_1 Atu3025. To confirm the result, more PL15 enzymes were selected for the construction of the phylogenetic tree, and, VBAly15A, together with other 35 enzymes from the CAZy database, form a single branch, which is apart from the PL15_1 family. Therefore, we proposed that VBAly15A and OalA belong to a new PL15 subfamily designed as the PL15_3 subfamily. Oals belonging to the PL15_3 subfamily are mainly from the *Vibrio* species (Fig. S1). Further structure comparison showed that compared to PL15_1 Oals, an N-terminal β-sheet in PL15_3 Oals is missing; instead, a α-helix is present in PL15_3 Oals. In addition, structural alignment revealed that the major difference between PL15 and PL17 Oals is also found in their N-terminal catalytic domain: the additional β-sheet present in the PL15_1 subfamily and the additional α-helix present in the PL15_3 subfamily, which are absent in PL17 Oals (Fig. S2). Taken together, our bioinformatic data suggested that VBAly15A from *Vibrio* sp. B1Z05 belongs to a new PL15 subfamily, PL15_3.

### VBAly15A is a medium–low temperature, alkaline, and polyM-specific Oal

### Protein expression and purification

**Fig 2:**
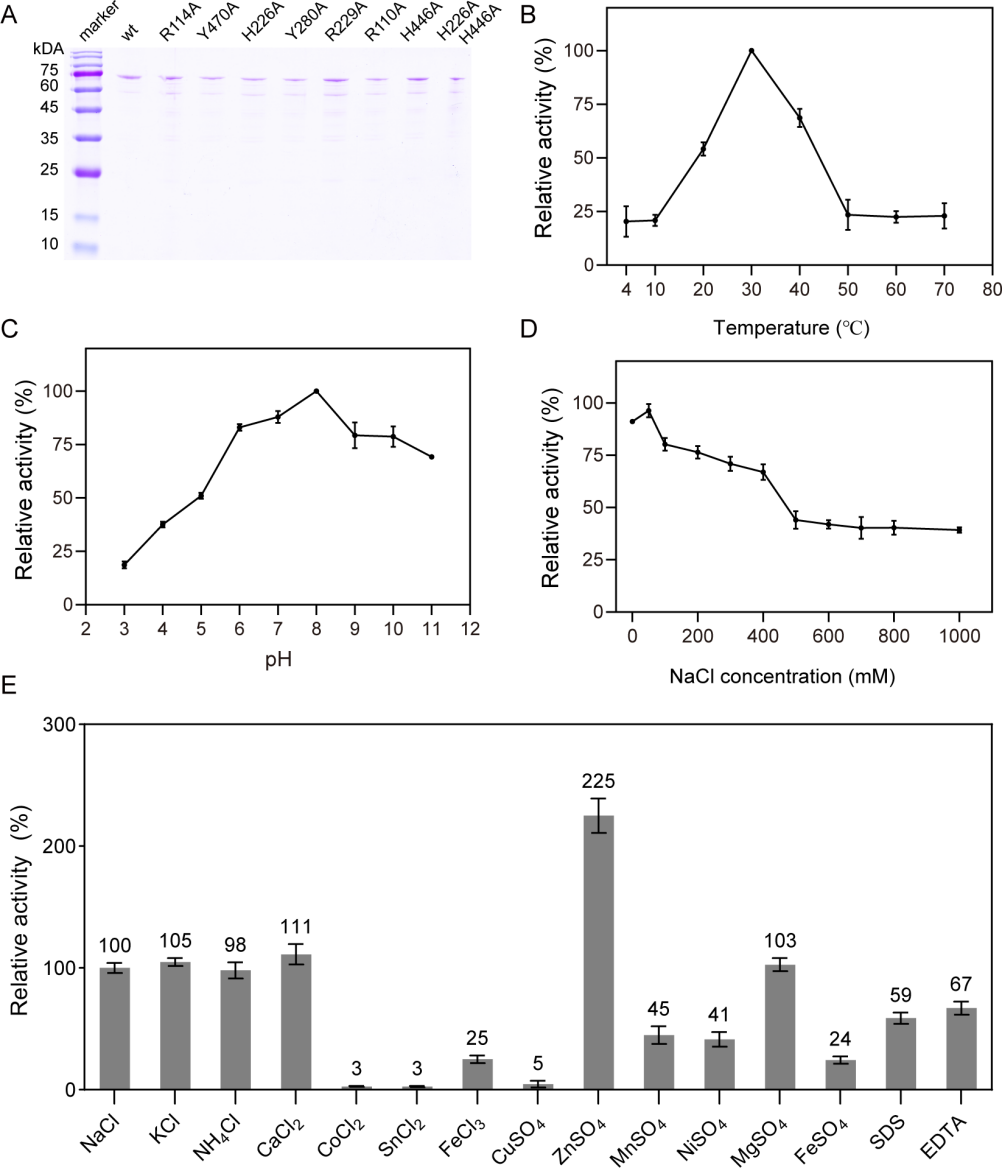
Effects of different enzymatic reaction conditions on the activity of VBAly15A toward sodium alginate. (**A**) SDS-PAGE analysis of purified VBAly15A and its mutants. VBAly15A protein with the molecular weight of about 79.82 kDa. (**B**) Optimal catalytic temperature of VBAly15A. (**C**) Optimal catalytic pH of VBAly15A. (**D**) Optimal NaCl concentration for VBAly15A activity. (**E**) Effect of chemical compounds on the activity of VBAly15A. The results from representative experiments were examined in triplicate. Values are given as the means ± standard deviations.

To understand its function, VBAly15A was expressed in *Escherichia coli* strain BL21 (DE3) and purified with Ni-NTA affinity chromatography. The molecular weight of VBAly15A on the SDS-PAGE was consistent with its theoretical value of 79.82 kDa (Fig. 2A). The protein yield was about 48 mg per 1 L LB culture. The size exclusion chromatography suggested that VBAly15A acts as a monomer in buffer since only a single peak was observed, with a molecular weight of 79.82 kDa (Fig. S3). Similarly, the PL15 Oal Atu3025 from *A. tumefaciens* strain C58 also migrates as a monomer in the size exclusion chromatography (28). However, the PL15 OalA from *V. splendidus* 12B01 was observed to be a homodimer (29).

### Effect of temperature and pH

VBAly15A displayed the maximum activity at 30°C, which was obviously reduced at 20°C or 40°C. When the temperature was below 10°C or above 50°C, only less than 25% activity was retained (Fig. 2B). When measured at different pH conditions, VBAly15A showed the greatest activity at pH 8.0, and a slight decrease was observed when pH values were higher than 9.0. VBAly15A is more sensitive to acid conditions, and when pH was lower than 5, 50% activity was retained (Fig. 2C). Therefore, VBAly15A is medium–low temperature and alkaline. This is different from its closest enzyme, OalA, which showed the greatest activity at pH 6.5 at 16°C (29).

### Effect of metal ions and other chemical compounds

Since a lot of extracellular alginate lyases identified from marine microorganisms are usually Na^+^-dependent (15), we wondered whether the activity of VBAly15A located in the cytoplasm was affected by NaCl. To this end, the activity of VBAly15A was examined under different NaCl conditions. As shown in Fig. 2D, NaCl was not required for the activity of VBAly15A, and the enzyme exhibited the highest activity when a trace amount of NaCl (50 mM) was present. The activity of VBAly15A decreased with increasing concentrations of NaCl, and less than 40% of activity was retained when the NaCl concentration was higher than 500 mM (Fig. 2D). Although five PL15 Oals have been identified, their optimal NaCl concentration remains unknown (Table S1). In addition, the effect of other metal ions at a concentration of 1 mM was also tested. As shown in Fig. 2E, the activity was significantly decreased in the presence of Fe^2+^, Fe^3+^, Ni^2+^, and Mn^2+^, and Cu^2+^, Sn^2+^, and Co^2+^ fully inhibited the activity of VBAly15A. No obvious difference in enzymatic activity was observed when Mg^2+^, Ca^2+^, K^+^, or NH_4_^+^ was used instead of Na^+^. Zn^2+^ could significantly increase the activity of VBAly15A, which was completely different from the PL15 Oal Atu3025 from *A. tumefaciens* strain C58, and the activity of Atu3025 was 100% inhibited by the same amount of Zn^2+^ (28).

### Specific activities toward different alginate substrates

**Fig 3:**
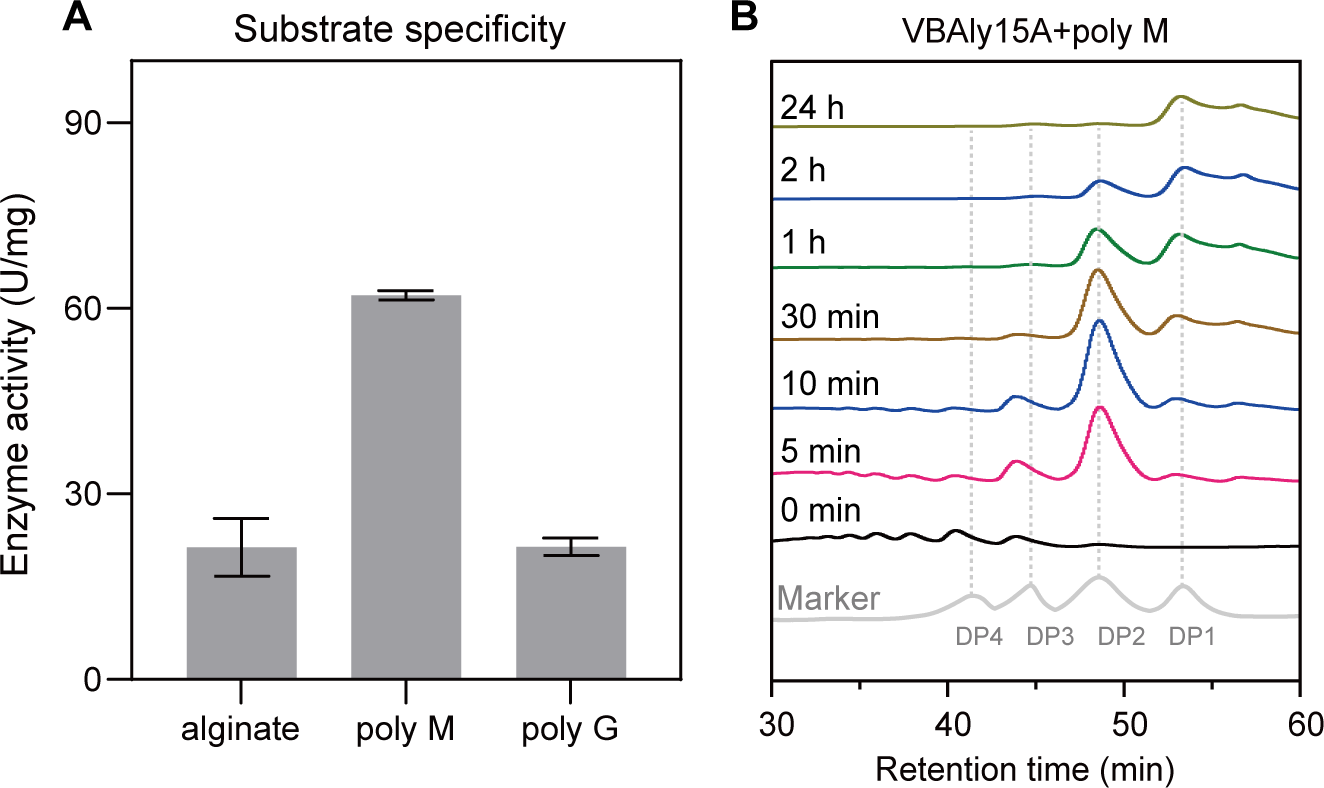
Substrate specificity and action mode of VBAly15A. (**A**) Substrate specificity of VBAly15A toward sodium alginate, polyM, and polyG. The results from representative experiments were examined in triplicate. Values are given as the means ± standard deviations. (**B**) The HPLC results show the product formation at different time points. PolyM is used for the product analysis. The results are obtained from representative experiments.

VBAly15A showed the greatest activity toward polyM, with a specific activity of 62.143 ± 0.737 U/mg (Fig. 3A). When sodium alginate or polyG was used as the substrate, the activity of VBAly15A was significantly reduced, with specific activities of 21.375 ± 4.676 and 21.447 ± 1.394 U/mg, respectively. These data indicated that compared to sodium alginate and polyG, VBAly15A prefers to use polyM as the substrate.

### Action mode and degrading products

The unsaturated sugars produced by VBAly15A were examined by high-performance liquid chromatography (HPLC) over time. At the beginning of the reaction, the major products were unsaturated disaccharides, which were gradually accumulated until the 10 min reaction (Fig. 3B). During these times, only a small number of unsaturated monomers (Δ) were observed. After 1 h of reaction, the amount of unsaturated disaccharides decreased, while the amount of unsaturated monomers increased. Based on the fact that disaccharides were gradually accumulated, and only unsaturated sugar could be detected by UV at 235 nm, VBAly15A is proposed to digest the substrate from the nonreducing end of the sugar chain in an exolytic mode (Fig. S4). It appears that only when substrates were fully converted into disaccharides did VBAly15A start to convert disaccharides into unsaturated monomers. The final amount of unsaturated monomers was slightly reduced due to the conversion of unsaturated monomers to DEH, a typical trait of exolytic alginate lyase. Taken together, our data suggested that when polymer alginates are used as the substrate, VBAly15A first degrades polymers into disaccharides, and then catalyzes disaccharides into monomers in an exolytic mode.

### A synergy occurs between VBAly15A and PL17 Oal

**Fig 4:**
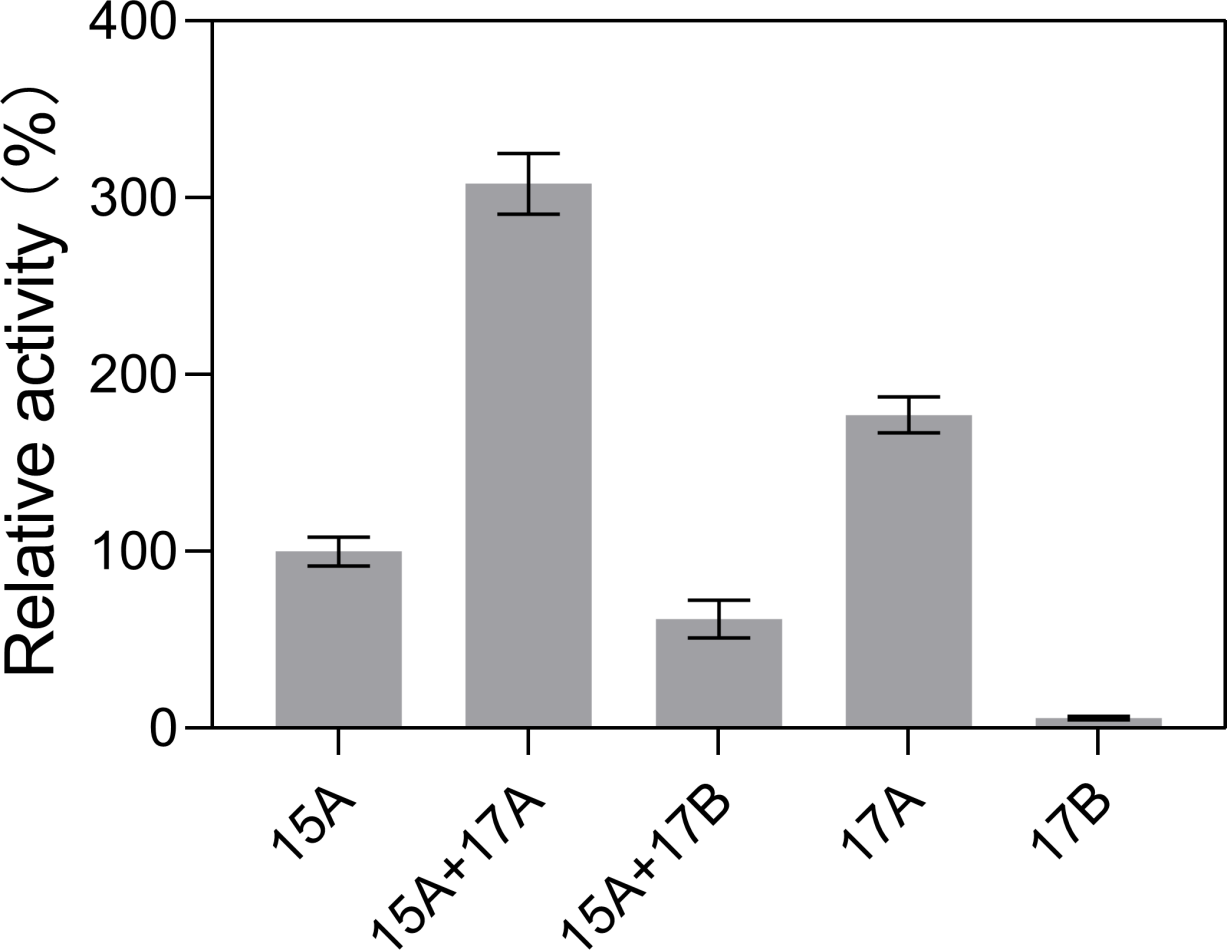
Synergistic effect assessed by mixing VBAly15A separately with *Va*Aly17A and *Va*Aly17B. Results are based on triplicate experiments and presented as mean ± standard deviation.

Our previous study showed that in alginate-degrading *Vibrio* species that only contain a pair of PL17 Oals, two PL17 Oals play a different role in AOS decomposition: one is involved in the conversion of larger AOSs into disaccharides, and the other is specific to digesting disaccharides into monomers. However, VBAly15A *per se* could achieve a full degradation of larger AOSs into monomers. Therefore, we wondered whether VBAly15A and the PL17 Oal pair could synergistically digest alginate. To verify this, a PL17 Oal pair (*Va*Aly17A and *Va*Aly17B) from *Vibrio alginolyticus* ATCC 17749 that catalyzes the substrate from the reducing site was used for the synergy of VBAly15A and the PL17 Oal pair. As shown in Fig. 4, when VBAly15A was mixed with *Va*Aly17A (which only degraded larger AOSs into disaccharides) at a 1:1 molar ratio, the enzyme activity toward sodium alginate was 208% and 57% higher than those of VBAly15A and *Va*Aly17A, respectively. The activity of the mixture of VBAly15A and *Va*Aly17B (which only degraded disaccharides into monomers) was 38% lower than that of VBAly15A, which is likely caused by the weak activity of *Va*Aly17B. But, even the total activity of the mixture of VBAly15A and *Va*Aly17B was still 6% higher than the sum activity of two proteins alone, indicating that a synergy also occurs between VBAly15A and *Va*Aly17B. This synergy may be due to a different degrading direction, with VBAly15A digesting the substrate from the nonreducing end of the sugar chain and *Va*Aly17A and *Va*Aly17B degrading the substrate from the reducing end. Taken together, our data suggested that VBAly15A could cooperate with *Va*Aly17A to rapidly degrade AOSs.

### Amino acid residues Arg^114^, Tyr^470^, and Arg^110^ are essential for the stable binding of the substrate

**Fig 5:**
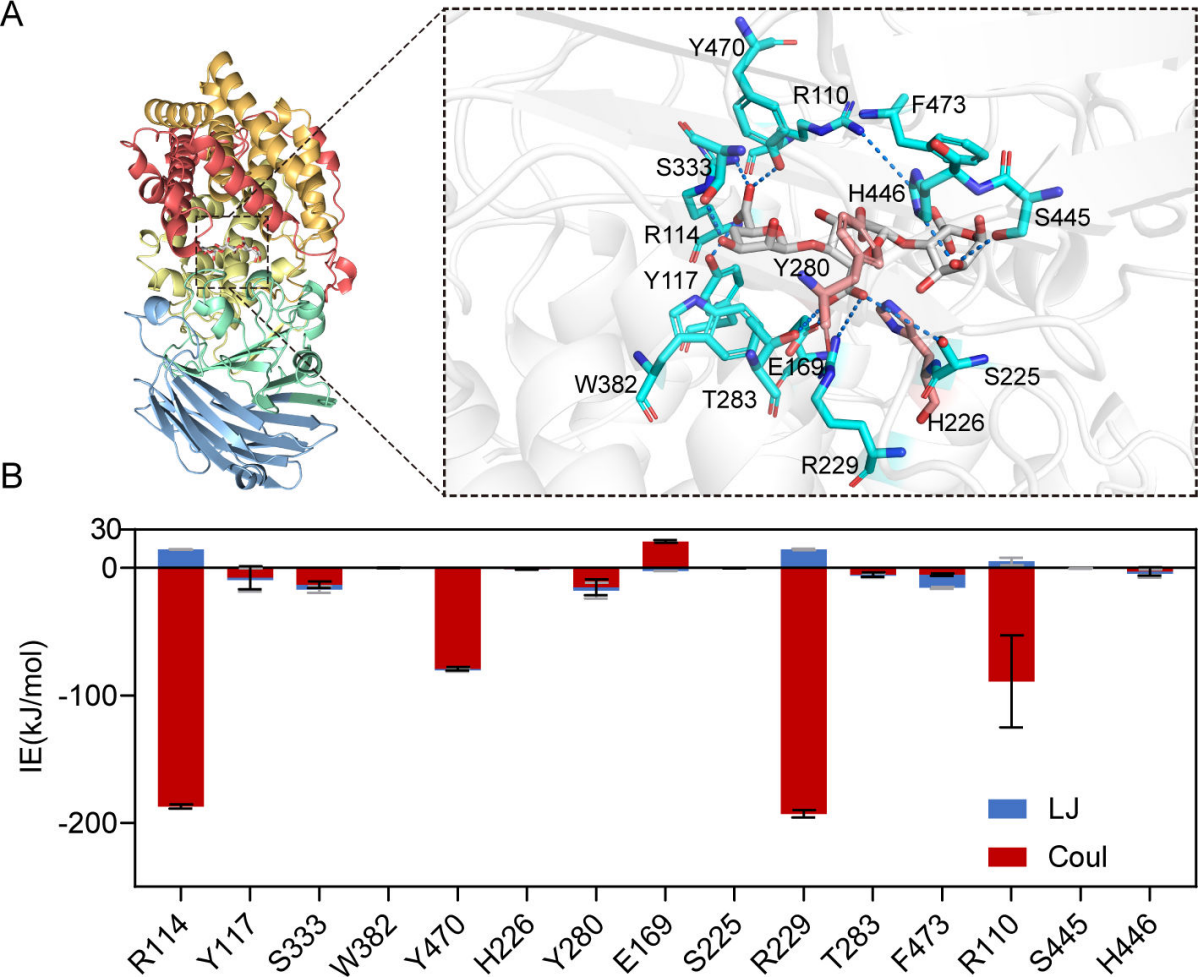
Activity structure analysis of VBAly15A. (**A**) Structure analysis of VBAly15A. The white represents the substrate sugar ring; the blue indicates the amino acids within a 5 Å radius; and the pink highlights the catalytic residues. (**B**) Molecular dynamics simulation of VBAly15A. The interaction energies between the amino acids within a 5 Å radius of the VBAly15A substrate were calculated. LJ represents van der Waals forces, and Coul represents Coulombic forces, both calculated using the energy tool in the GROMACS package.

**Fig 6:**
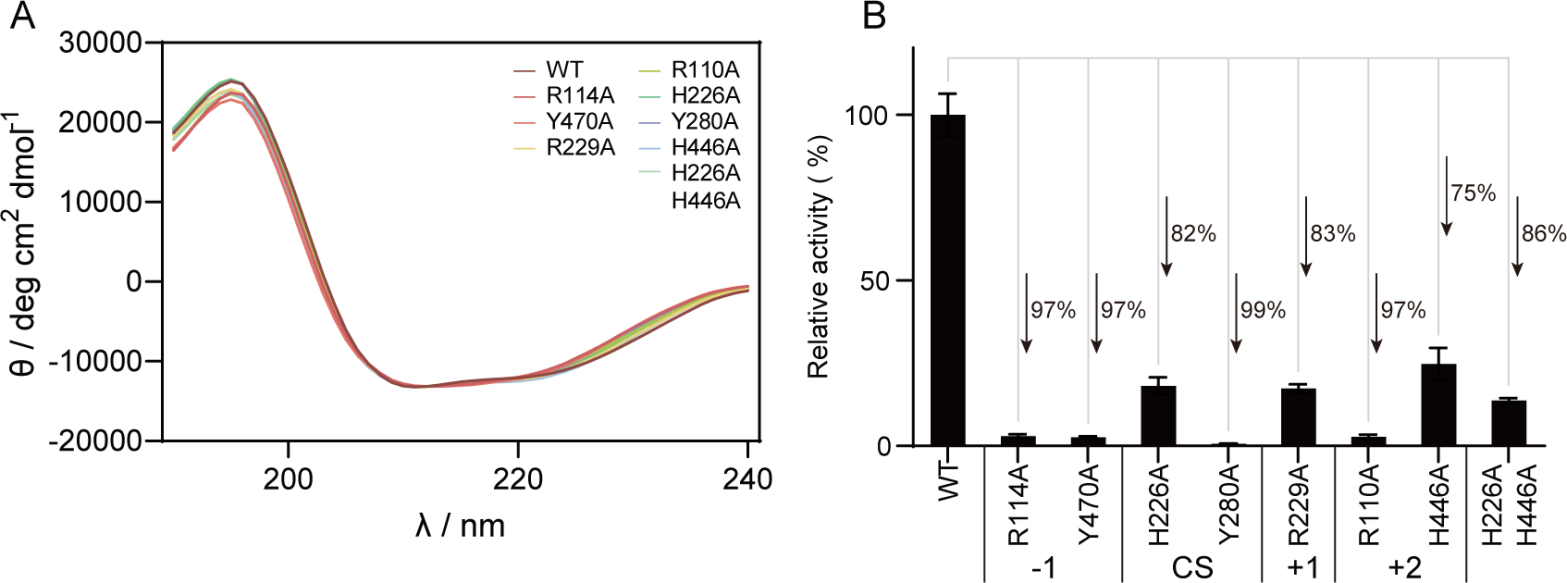
Effect of different residue mutations on the protein structure and alginolytic activity. (**A**) Circular dichroism spectra of WT and mutants. (**B**) Enzyme activities of different mutants toward sodium alginate. Results are based on triplicate experiments and presented as mean ± standard deviation.

To better uncover its catalytic mechanism, the VBAly15A–uMGG complex was constructed by aligning VBAly15A with the complex of Atu3025–uMGG (PDB: 3AFL; uMGG, unsaturated trisaccharide) to obtain the sequence composition of the active center of VBAly15A (Fig. 5A). According to the observation of the complex of Atu3025–uMGG (PDB: 3AFL) (31), amino acid residues His^226^ and Tyr^280^ in VBAly15A were proposed to function as the catalytic base and the catalytic acid of the β-elimination reaction, respectively. The protonated residue Glu^169^ and the residue Arg^229^ are required to neutralize the negative charge on the carboxyl at the +1 subsite, which are universal and conserved in several alginate lyase families (19, 33). Further molecular dynamics simulations of the complex of VBAly15A–uMGG suggested that Arg^114^, Tyr^470^, Arg^229^, and Arg^110^ have strong hydrogen bond interactions with the substrate (Fig. 5B), which may be essential for substrate binding. To verify their role in catalytic activity, four mutations, namely, R114A, Y470A, R229A, and R110A, were constructed and purified (Fig. 2A), and circular dichroism spectra confirmed that the mutants do not have obvious changes in the main chain structures compared to the wild-type (WT) protein (Fig. 6A). In agreement with the data from molecular dynamics simulations, the activities of four alanine substitutions toward sodium alginate were dramatically reduced (Fig. 6B). Among them, only R229A binding the sugar at the +1 subsite still retained 17% of the activity of the WT because Glu^169^ at the +1 subsite could partially complement the catalytic efficiency. However, little alginolytic activity was observed in the other mutants that have interactions with sugars at the −1 (R114A, Y470A) and +2 subsites (R110A) (Fig. 6B). Therefore, Arg^114^, Tyr^470^, and Arg^110^ in VBAly15A are essential for the stable binding of the substrate. In addition, Arg^114^, Tyr^470^, and Arg^110^ are fully conserved in all identified PL15 Oals (Fig. S5). Therefore, these three residues in the PL15 family of Oals may have a similar function to that in VBAly15A and be required for substrate binding of the PL15 Oals.

### Candidate catalytic base His^226^ is not fully required for the β-elimination reaction

**Fig 7:**
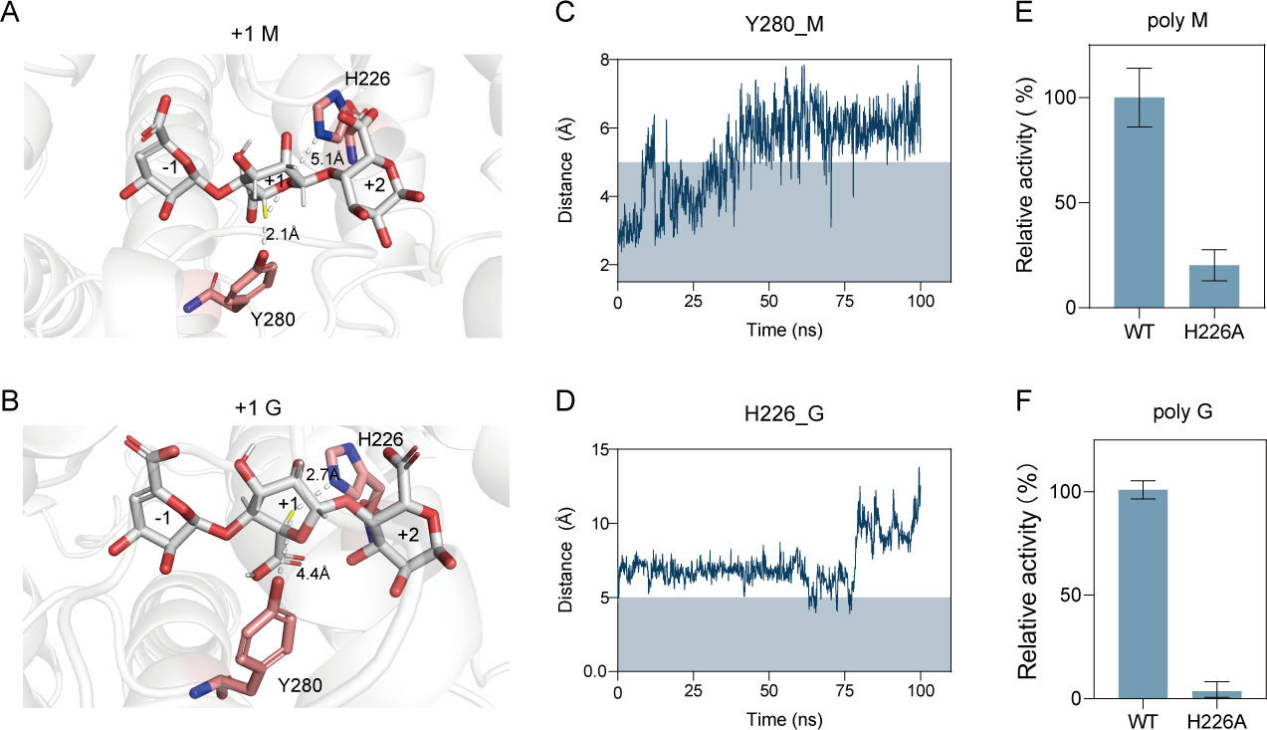
Interaction analysis between catalytic residues of VBAly15A and substrates. (**A**) Distance between the catalytic residue and the C5 proton at the +1 position of the M-type substrate. (**B**) Distance between the catalytic residue and the C5 proton at the +1 position of the G-type substrate. (**C**) Distance fluctuations between Tyr^280^ and the C5 proton at the +1 position of the M-type substrate during a 100 ns molecular dynamics simulation. (**D**) Distance fluctuations between His^226^ and the C5 proton at the +1 position of the G-type substrate during a 100 ns molecular dynamics simulation. (**E**) Enzymatic activity of the H226A mutant toward polyM. (**F**) Enzymatic activity of the H226A mutant toward polyG. Results are based on triplicate experiments and presented as mean ± standard deviation.

In addition to the aforementioned residues, the roles of residues His^226^ and Tyr^280^ involved in alginate catalysis were also investigated by site-directed mutation. Unexpectedly, although the Y280A mutant did not show activity toward sodium alginate, H226A still maintained 18% of the WT activity (Fig. 6B), indicating that only Tyr^280^, but not His^226^, is required for the β-elimination reaction. On the contrary, an additional His^446^ at the +2 subsite is present around the active groove (Fig. 5A), which is suggested to be involved in the catalysis in the PL15_2 heparinase subfamily (34). Based on this, we first hypothesized that His^446^ in VBAly15A might also function as a catalytic base for alginate degradation. However, neither the H446A single mutant nor the H226A/H446A double mutant completely lost their catalytic activities toward sodium alginate (Fig. 6B), suggesting that His^446^ in VBAly15A may not act as a catalytic base but function for substrate binding. To uncover the catalytic mechanism of VBAly15A, a close-up view of the interaction between VBAly15A and the substrate was further analyzed. As shown in Fig. 7A, the distances of the residue His^226^ to the proton of the C5 in the M- (uMMG) and G-type (uMGG) trisaccharides at the +1 subsite (where the catalytic base could abstract the proton and the double-bond forms between C4 and C5) are 5.1 and 2.7 Å, respectively, whereas the distances between Tyr^280^ and the C5 proton of uMMG and uMGG are 2.1 and 4.4 Å (Fig. 7B), respectively. On the contrary, Tyr can act as both a catalytic base and a catalytic acid in the PL5 alginate lyase (35–39), and the retained 18% of the WT activity in the VBAly15A H226A mutant may be due to the redundant function of Tyr^280^. Moreover, the observation that Tyr^280^ has a lower distance with the C5 proton of the type M sugar compared to the amino acid His^226^ might result in the polyM-specific activity. In line with this, molecular dynamics simulations showed that the distance between Tyr^280^ and the C5 proton of the M-type sugar at the +1 subsite was much shorter than that between His^226^ and the G-type sugar (Fig. 7C and D). Based on this, it was proposed that Tyr^280^ may act as both the catalytic acid and the catalytic base when polyM is used as the substrate, and the substrate specificity of VBAly15A may be determined by the distance between the catalytic residue and the C5 proton at the +1 position of the substrate. Consistent with this, the activity of H226A toward polyG was hardly observed, and about 20% activity was retained when polyM was used as the substrate (Fig. 7E and F). To investigate whether the fact that the distance of His^226^ and Tyr^280^ in the active site of VBAly15A is closely related to substrate specificity is common in the PL15 Oals, we modeled all characterized PL15 family alginate lyases and measured the distances between the catalytic residues and the C5 proton at the +1 position of the substrate. Our data showed that the substrate specificities of these identified PL15 Oals could be determined by the distance of the catalytic residues His and Tyr to the C5 proton of the sugar ring at the +1 subsite: if His is farther from the C5 proton of the G ring at the +1 subsite compared to the distance between Tyr and the C5 proton of the M ring, the enzyme will exhibit polyM specificity (Table 1).

**TABLE 1:** Distance between catalytic residues and substrate in PL15 family Oals and their substrate specificity^*a*^

Name	Base	Dist. (Å)	Base/acid	Dist. (Å)	Substrate specificity	Source or reference
VBAly15A	H226	M 5.1	Y280	**M 2.1**	**Poly M**	This study
		**G 2.8**		G 4.4		
VSAly15A	H282	M 4.6	Y337	**M 1.6**	**Poly M**	Unpublished data
		**G 2.4**		G 3.9		
VSAly15B	H226	M 5.0	Y280	**M 2.1**	**Poly M**	Unpublished data
		**G 2.7**		G 4.5		
A1-IV	H296	M 4.5	Y350	**M 1.6**	**Poly M**	(26)
		**G 2.3**		G 4.0		
A1-IV'	H240	M 4.0	Y293	**M 1.6**	**Poly M**	(27)
		**G 1.8**		G 1.8		
Atu3025	H311	M 4.3	Y365	**M 1.9**	**Poly M**	(28)
		**G 2.2**		G 4.3		
OalA	H226	M 4.8	Y280	**M2.3**	**Poly M**	(29)
		**G 2.6**		G4.7		
AlyPB2	H214	M 4.6	Y268	**M 2.0**	**Poly M**	(30)
		**G 2.3**		G 4.6		

^*a*^ Bold text is used to highlight the parts with shorter distances and those related to substrate specificity.

## DISCUSSION

Alginates are the most abundant polysaccharides present in the cell walls of brown macroalgae. Although the composition of alginate is rather simple and only consists of two conformational isomers, complex systems are required for its full degradation. During this process, alginate lyases need to recognize their preferred substrates, and even within the same PL family, alginate lyases exhibit different specific catalytic mechanisms. However, little is known about the key factor determining substrate specificity. In this work, we identified a novel Oal VBAly15A that belongs to a new subfamily of PL15, PL15_3. The major difference between PL15_1 and PL15_3 is their N-terminal structure: a β-sheet in PL15_1 and a long α-helix in PL15_3. In addition, PL15_2 is a heparinase subfamily that was first identified by Zhang et al. (34). The characterized PL15_2 exolytic heparinase, BIexoHep from *Bacteroides finegoldii* DSM 17565, shows a similar protein structure to the PL15_1 Oal Atu3025, while its catalytic amino acid residues (His^337^, Tyr^390^, and His^555^) are distinct from those in Atu3025 (His^311^ and Tyr^365^) (34). In line with this, the active site of VBAly15A only contains two catalytic residues (His^226^ and Tyr^280^). The residue His^446^ situated at an equivalent position to His^555^ in BIexoHep shows a hydrogen bond interaction with the sugar ring at the +2 subsite.

Different from the catalytic acid Tyr^280^, the H226A mutant still retained 18% of the activity of the WT when sodium alginate was used as the substrate. This result indicated that the catalytic base His^226^ in VBAly15A is not fully required for the degradation of sodium alginate. Alternatively, Tyr^280^ may serve as both the catalytic acid and the catalytic base. In line with this, another (α/α)_n_ toroid-type alginate lyase family, PL5, does not contain an additional catalytic base at the active site for G-type substrates and thus could only digest M-type substrates (35–39). Therefore, His^226^ in VBAly15A previously proposed to act as a catalytic base may not be required for all enzyme activities. In some alginate lyases, Tyr^280^ can function as both the catalytic acid and the catalytic base. Further structural analysis revealed that the polyM-specific catalysis of VBAly15A is likely due to a shorter distance between Tyr^280^ and the C5 proton of the M-type sugar ring compared to that between His^226^ and the C5 proton of the G-type sugar. The observation seems to extend to all identified PL15 alginate lyases. For example, in PL15 alginate lyase Atu3025 from *A. tumefaciens* strain C58, the distance between Tyr^365^ and the C5 proton of the M-type substrate is shorter than that between His^311^ and the C5 proton of the G-type substrate, leading to a higher activity toward polyM compared to that toward polyG (28). Moreover, a His mutation (H531A) in Atu3025 led to a significantly reduced activity toward uGGG, thereby resulting in the crystal structure of the H531A–uGGG complex obtained (31). The observed feature about the distance between the catalytic base and the substrate appears not to be limited in the PL15 Oals. For example, in the PL39 alginate lyase DP0100 from *Defluviitalea phaphyphila*, His^187^ has a shorter distance with the C5 proton of polyG compared to that between Tyr^239^ and the C5 proton of polyM (Fig. S6), so DP0100 is identified as polyG-specific (40). In the PL17 Oal Alg7c from the marine bacterium *Saccharophagus degradans*, the residue His^202^ located at the same site as the catalytic base His in PL15 was first hypothesized to be a catalytic base. However, when His^202^ was mutated into Ala, its value of *k_cat_*/*K_m_* indicated that His^202^ is not fully required for alginate degradation (41). The low activity of the H202A mutant in Alg7c may be due to the presence of the residue Tyr^258^, which could serve as both a catalytic base and a catalytic acid. In line with this, the H226A mutant in VBAly15A also retained 18 and 25% activities of the WT protein when sodium alginate and polyM were used as substrates, respectively. However, the activity of the H226A mutant toward polyG was hardly detected, implying that in VBAly15A, His^226^ may be specific for the degradation of polyG, while Tyr^280^ functions as both a base and an acid catalyst toward polyM. The substrate specificity is determined by the shorter distance between the catalytic base and the C5 proton. Recently, a novel polyG-specific PL7 alginate lyase OUC-FaAly7 was reported and showed a similar specific catalytic mechanism: the shorter distance between the catalytic base His^99^ and the C5 proton of the G-type substrate is a key factor in determining substrate specificity (42). Therefore, the distances of different catalytic bases to the C5 proton of the uronic acid moiety of their corresponding substrates are critical for enzyme substrate specificity.

### Conclusion

In summary, a novel Oal VBAly15A was identified, which belongs to a new subfamily of PL15, PL15_3. Biochemical characterization suggested that VBAly15A is medium–low temperature, alkaline, and polyM-specific. The residue Tyr^280^ in the active center site likely serves as both the catalytic base and the acid. In addition to Tyr^280^, the residue His^226^ could also abstract the proton, which is specific for the degradation of the G-type substrate. The distance of Tyr^280^ to the C5 proton of the mannuronic acid moiety at the +1 subsite is shorter than that of His^226^ to the C5 proton of the guluronic acid moiety, thereby leading to polyM specificity. The specific catalytic mechanism could extend to alginate lyases in the PL15 family. This work provides new insight into the specific catalytic mechanism of PL15 alginate lyases.

## MATERIALS AND METHODS

### Strains and materials

All strains used in this research are shown in Table S2. *E. coli* strains, including DH5α and BL21 (DE3), were obtained through Tsingke (Beijing, China), which were used as the plasmid cloning and protein expression hosts, respectively. *Vibrio* sp. B1Z05 isolated from the abalone gut (32) was used to obtain the Oal VBAly15A gene. *E. coli* strains were cultured in LB medium, and *Vibrio* sp. B1Z05 was cultured in 2216E medium at 28°C. When needed, 30 µg/mL of kanamycin was mixed into cultured *E. coli* cells. Sodium alginate (≥97% purity) was purchased from Sigma-Aldrich (St. Louis, MO, USA) with an M/G value of 1.56 (61/39) and a viscosity of 4–12 mPa⋅s. PolyG (≥97% purity) and PolyM (≥97% purity), having a degree of polymerization (DP) between 27 and 37, were obtained from BZ Oligo Biotech Co., Ltd. (Qingdao, China).

### Bioinformatics analysis

The *VBAly15A* gene (GenBank accession number: WP_023403303.1) was found in the genome of *Vibrio* sp. B1Z05 (GenBank accession number: GCF_009372095.1). The domain organization of VBAly15A was obtained by SMART (https://smart.embl.de) (43). The ExPASy server (https://www.expasy.org) maintained by the Swiss Institute of Bioinformatics was utilized to calculate the molecular weight (MW). The protein alignment was carried out using ClustalW (http://www.clustal.org/clustal2/) (44). The maximum likelihood estimation method was applied to construct the phylogenetic tree with FastTree, and 1,000 bootstrap replicates were performed (45). Alginate lyases for constructing the phylogenetic trees were downloaded from the CAZy database (http://www.cazy.org/) (46).

### Protein expression and purification

The plasmid pLYJ163 was used as the vector for protein heteroexpression. First, the *VBAly15A* gene was obtained from the genome of *Vibrio* sp. B1Z05, then ligated into the NcoI/XhoI-digested plasmid pLYJ163, yielding the plasmid pTYQ01. Site-directed mutation was achieved using a PCR-based method (47). All plasmids constructed in this work are listed in Table S2, and primers are shown in Table S3. The constructed plasmid was transformed into *E. coli* BL21 (DE3), and the cells were initially cultured in 1 L of LB medium containing 50 µg/mL kanamycin at 37°C. Upon reaching an OD_600 nm_ value of 0.8, cells were cultured at 16°C, and protein expression was induced by adding 0.1 mM isopropyl-β-D-thiogalactopyranoside. After a 20 h cultivation, cells were separated through centrifugation at 10,000 *g* at 4°C for 10 min, resuspended in the balance buffer (50 mM Tris–HCl, 150 mM NaCl, pH 8.0), and lysed by sonication in an ice bath. Following centrifugation, the supernatant was collected and loaded onto a Ni-NTA sepharose column (GE Healthcare, USA) to obtain the purified protein. Subsequently, a PD-10 column was used to eliminate imidazole, and SDS-PAGE was performed to confirm the enzyme. A HiLoad 16/600 Superdex 200 prep-grade column (GE Healthcare, USA) was used to understand the oligomeric state of VBAly15A. IgG (158 kDa), human albumin (66 kDa), ovalbumin (44 kDa), and myoglobulin (17 kDa) from GE Healthcare were used as protein size standards. The protein concentration was measured with a NanoPhotometer N60 (Implen, Germany).

### Enzyme activity assay and biochemical characterization

The activities of VBAly15A toward sodium alginate, polyM, and polyG were examined by using a UV spectrometry method (48). Briefly, 180 µL of the substrate solution (3 mg/mL, 50 mM Tris–HCl, 150 mM NaCl, pH 8.0) was fully mixed with 20 µL of enzyme. After incubation at 30°C for 10 min, the mixture was then incubated at 100°C for 10 min to terminate the reaction, followed by cooling to room temperature. Following this, 150 µL of the mixture was used to obtain the absorbance at 235 nm. One unit (U) of the enzyme was defined as the quantity needed to cause a 0.1/min increase in absorbance at A_235 nm_.

To determine the optimal reaction conditions, various buffers were prepared. Enzyme activity under different conditions was measured using UV absorption spectroscopy. The greatest activity was defined as 100%, and relative activity under different conditions was calculated accordingly. For the optimal temperature assay, the mixture of the enzyme and sodium alginate was reacted in 50 mM Tris–HCl (150 mM NaCl, pH 8.0) at different temperatures (4, 10, 20, 30, 40, 50, 60, and 70°C). For the optimal pH assays, different pH conditions (pH 3–11) were achieved by using the Britton–Robinson buffer (phosphoric, boric, and acetic acids, each at 0.04 M), and the reaction was performed at 30°C in the presence of 50 mM NaCl. The pH of the solution is adjusted by adding 0.2 M NaOH solution. To test the effect of the NaCl concentration on the enzyme activity, multiple NaCl concentrations between 0 and 1.0 M (0, 0.05, 0.1, 0.2, 0.3, 0.4, 0.5, 0.6, 0.7, 0.8, 0.9, and 1.0 M) were prepared in 50 mM Tris–HCl buffer (pH 8.0) at 30°C. The effects of different metal ions and other chemical compounds were investigated by measuring enzyme activities at 30°C in 50 mM Tris–HCl buffer (50 mM NaCl, pH 8.0) containing different chemical agents (K^+^, NH_4_^+^, Ca^2+^, Co^2+^, Sn^2+^, Fe^3+^, Cu^2+^, Zn^2+^, Mn^2+^, Ni^2+^, Mg^2+^, Fe^2+^, EDTA) at 1 mM.

To obtain the enzyme activities of different mutants, the release of reducing sugars from sodium alginate was monitored using the dinitrosalicylic acid (DNS) method as follows: 100 µL of substrate solution (3 mg/mL, 50 mM Tris–HCl, 50 mM NaCl, pH 8.0) was fully mixed with 100 µL of enzyme. After incubation at 30°C for 30 min, the reaction solution was fully mixed with 100 µL of DNS solution, incubated at 100°C for 10 min, and cooled to room temperature. Following this, 200 µL of the mixture was used to obtain the absorbance values at 540 nm. Wild-type enzyme activity is defined as 100% to calculate the enzyme activities of different mutants.

For the synergy assay, a pair of PL17 Oals *Va*Aly17A and *Va*Aly17B from *V. alginolyticus* ATCC 17749 was used. In the experiment, each enzyme was used at the same molar concentration (12.53 µM). Each enzyme was either added individually (100 µL VBAly15A, 100 µL *Va*Aly17A, 100 µL *Va*Aly17B) or in pairs (50 µL VBAly15A + 50 µL *Va*Aly17A, 50 µL VBAly15A + 50 µL *Va*Aly17B) to 100 µL of a 3% alginate solution. The mixture was then incubated at 30°C for 30 min, after which enzyme activity was measured using the DNS method.

### Degrading product analysis

The sugar products produced by VBAly15A toward sodium alginate were examined through HPLC. In brief, the reaction mixture, in which 1.5 mg/mL enzyme protein and 3 mg/mL sodium alginate were added, was placed in 50 mM Tris–HCl buffer (pH 8.0, 150 mM NaCl) at 30°C for different time intervals. After termination at 100°C for 10 min, the sample was filtered through 0.22 µm filters and loaded to a Superdex peptide 10/300 GL gel filtration column (GE Healthcare, Madison, USA) with a flow rate of 0.3 mL/min. The degrading products were detected by examining the absorbance at 235 nm.

### Circular dichroism spectra

The main chain structural changes of the WT protein and its mutants were tested by circular dichroism spectra (49). Different proteins were dissolved in 200 µL of phosphate-buffered solution to a concentration of 0.1 mg/mL. The sample was detected in a J-1500 CD spectrophotometer (JASCO, Tokyo, Japan), with the temperature set to 25°C, the scanning rate at 200 nm/min, and the path length of 0.1 cm. The data were collected at wavelengths ranging from 190 to 240 nm.

### Structure prediction, comparison, and analysis

Protein structures were predicted by AlphaFold2 (50), and the open source code is obtained from GitHub (https://github.com/deepmind/alphafold). The pLDDT values were used as criteria for the structure selection. Structure visualization and alignment were performed on PyMOL Version 2.1.1. The substrates uMGG and uMMG were obtained from the complexes of Atu3025 (PDB: 3AFL) and Alg17C (PDB: 4OJZ), respectively. The protein–sugar complex was constructed by aligning the protein with a crystal structure, for example, constructing the complex of the PL5 enzyme Smlt1473–uMGG, a crystal complex of Atu3025–uMGG (PDB: 3AFL). Then, the protein of Atu3025 was hidden on PyMOL; the complex Smlt1473–uMMG was obtained; and the substrate was fine-tuned to ensure the same spatial position of the substrate at the +1 subsite. The complex was also examined by molecular dynamics simulation to confirm that the structure is stable.

### Molecular dynamics simulation

The CHARMM-GUI Solution Builder Module was used to construct the system, with the TIP3P water model employed for solvation. To neutralize the system, 150 mM NaCl was added, and the pH was set to 7.0. Molecular dynamics simulations were carried out with the GROMACS 2021.06 software package. Energy minimization of the system was conducted using the steepest descent method. During the equilibration phase, the system temperature was maintained at 313 K using the Nose–Hoover thermostat. Each simulation system was performed for 100 ns with the leapfrog algorithm using a time step of 2 fs. Long-range electrostatic interactions were handled using the PME algorithm, while the short-range neighbor list cutoff and short-range Coulomb cutoff radii were set to –1.2 nm. The LINCS algorithm was applied to constrain all hydrogen-containing bonds in the protein. Trajectory snapshots were captured at 0.5 ns intervals using the gmx trjconv tool and output as PDB files. Structural visualization was performed using PyMOL. Interaction energies were calculated using the gmx energy tool, and distance changes during the simulation were determined with the gmx distance tool.

## Data Availability

The protein sequences of VBAly15A, VaAly17A, and VaAly17B are available from the GenBank database under accession numbers WP_023403303.1, AGV20268.1, and AGV20269.1, respectively. In addition, all data supporting the findings of this study are available within the paper (and its supplemental material). All relevant data generated during this study or analyzed in this published article (and its supplemental material) are available from the corresponding author upon reasonable request.

## SUPPLEMENTAL MATERIAL

Supplemental material aem.02351-24-s0001.docx Figures S1 to S6; Tables S1 to S3.

ASM does not own the copyrights to Supplemental Material that may be linked to, or accessed through, an article. The authors have granted ASM a non-exclusive, world-wide license to publish the Supplemental Material files. Please contact the corresponding author directly for reuse.
