# Supplementary material for: Insights into the mechanism of substrate specificity in a novel PL15_3 subfamily oligo-alginate lyase VBAly15A
Source: Appl Environ Microbiol. 2025 Feb 27;91(3):e02351-24. doi: 10.1128/aem.02351-24 (PMC11921355; doi:10.1128/aem.02351-24)
Supplement: Supplemental material — Figures S1 to S6; Tables S1 to S3. [file aem.02351-24-s0001.docx]

Insights into the Mechanism of Substrate Specificity in a Novel PL15_3 Subfamily Oligo-Alginate Lyase VBAly15A

Yongqi Tang^a^, Ziyan Song^a^, Xiaodong Xu^b^, Yingjie Li^a#^, Lushan Wang^a^

^a^State Key Laboratory of Microbial Technology, Shandong University, Qingdao, China

^b^Qingdao Vland Biotech Company Group, Qingdao 266061, China

Running Head: Mechanism of Substrate Specificity in VBAly15A

#Address correspondence to Yingjie Li, yingjie.li@sdu.edu.cn


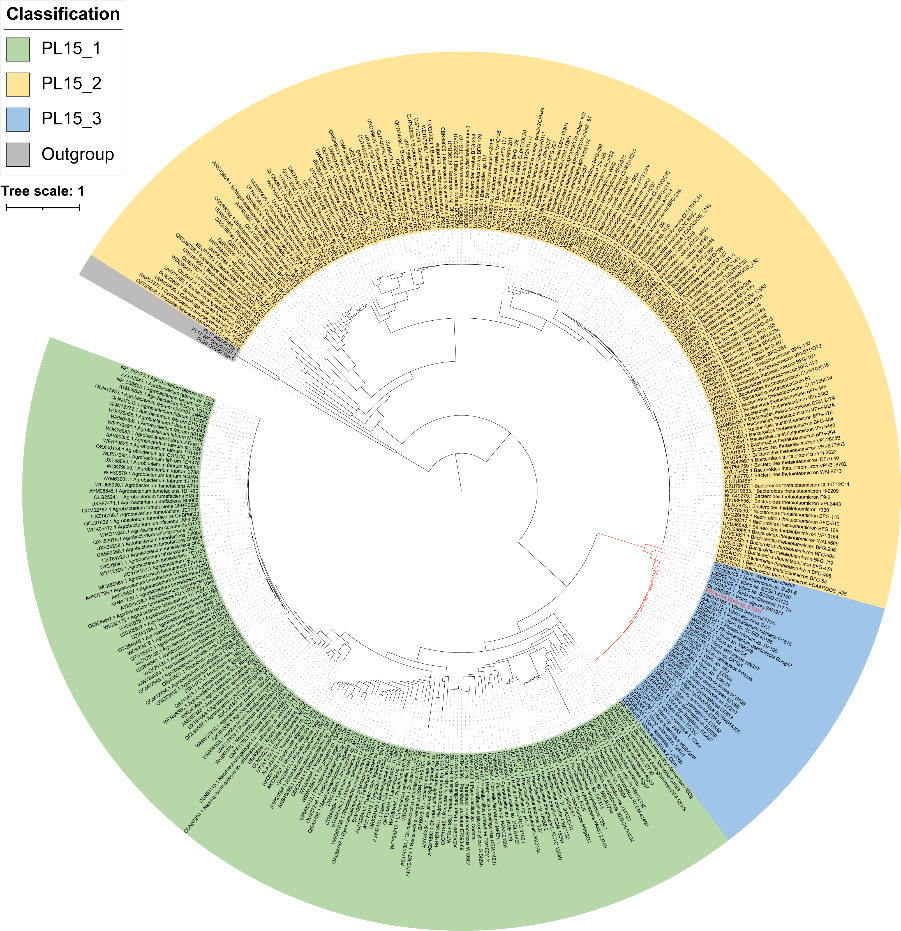


Figure S1. Phylogenetic analysis of the PL15 family: The protein alignment was carried out using ClustalW. The maximum likelihood estimation method was used to construct the phylogenetic tree on the Fast Tree, and 1,000 times of bootstrapping analysis was conducted.


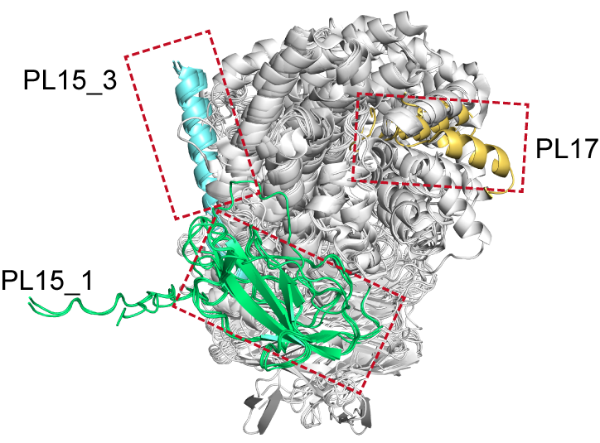


Figure S2. Structural comparison of alginate lyases from the PL15 and PL17 families. Green indicates the N-terminal β-sheet of the PL15_1 subfamily, blue indicates the N-terminal α-helix of the PL15_3 subfamily, and yellow indicates the N-terminal α-helix of the PL17 family.


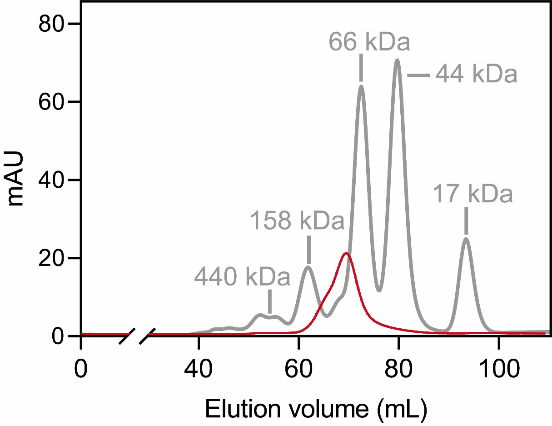


Figure S3. Size-exclusion analysis of the oligomeric state of VBAly15A in solution using myoglobulin (17 kDa), ovalbumin (44 kDa), human albumin (66 kDa), and IgG (158 kDa) as protein size standards.


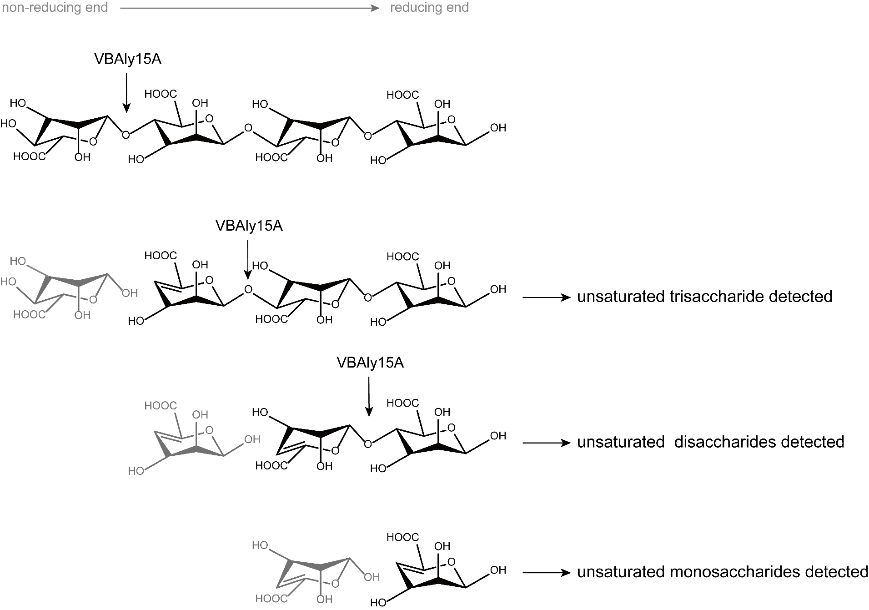


Figure S4. Degradation pattern of VBAly15A.


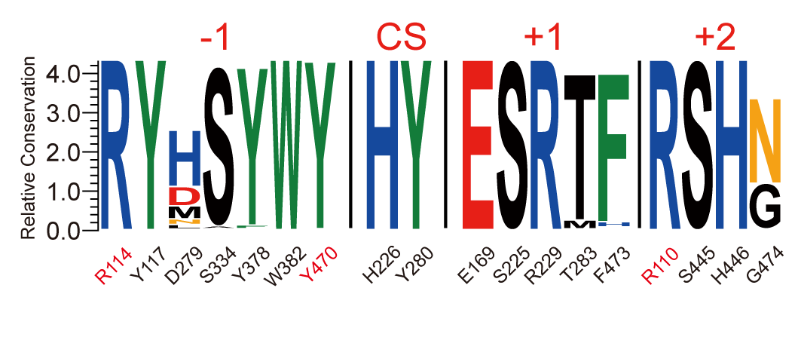


**Figure S5**. Conserved residue analysis of Arg^114^, Tyr^470^, and Arg^110^ in the PL15 family. CS indicates the catalytic site.


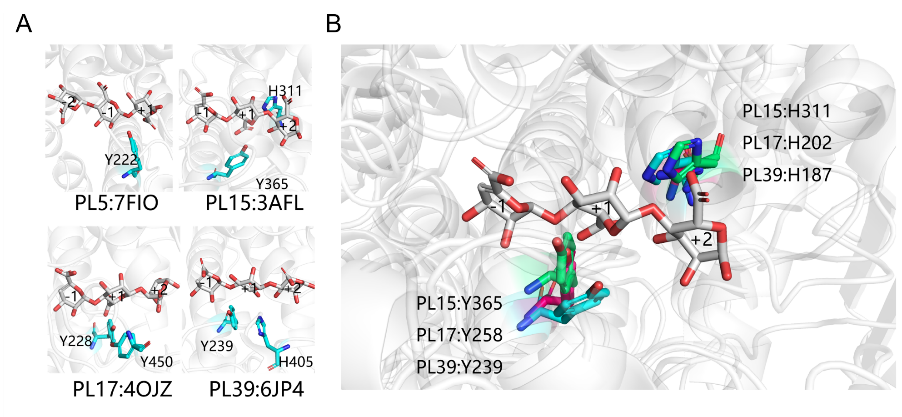
Figure S6. Structural comparison of catalytic residues in alginate lyases. (A) Catalytic residue composition in four families. (B) Structural superposition of the PL15, PL17, and PL39 families, with a prediction of the potential catalytic base site.

| Name | Subfamily | Temp (^o^C) | pH | [NaCl] (M) | Action mode | Substrate preference | End products (DP) | Microorganism | Reference |
| --- | --- | --- | --- | --- | --- | --- | --- | --- | --- |
| VSALy15A | PL15_3 | 30 | 8 | 0.05 | exolytic | polyM | 1 | *Vibrio* sp. B1Z05 | This study |
| Atu3025 | PL15_1 | 30 | 7.3 | NA | exolytic | polyM | 1 | *A. tumefaciens* strain C58 | (1) |
| AlyPB2 | PL15_1 | 20 | 8 | NA | exolytic | polyMG | 1 | *Photobacterium* sp. FC615 | (2) |
| A1-IV’ | PL15_1 | 30–37 | 7.5–8.5 | NA | endolytic | polyM | 2–3 | *Sphingomonas* sp. A1 | (3) |
| AlyFRB | PL15_1 | 25–35 | NA | NA | exolytic | polyM | 1 | *Falsirhodobacter* sp. alg1 | (4) |
| OalA | PL15_3 | 16 | 6.5 | NA | exolytic | polyM | 1,2 | *Vibrio splendidus* 12B01 | (5) |

**Table S1 Biochemical characteristics of PL15 alginate lyases**

**Table S2 Strains and plasmids used in this study**

| **Strain or plasmid** | **Description** | **Source or reference** |
| --- | --- | --- |
| **Strains** |  |  |
| *Vibrio alginolyticus* ATCC 17749 | Wild-type | (6) |
| *Vibrio* sp. B1Z05 | Wild-type | (7) |
| *E. coli* DH5α | The host strain used for general cloning | Dingguo (China) |
| *E. coli* BL21(DE3) | The host strain used for recombinant protein production | Tsingke (China) |
| **Plasmids** |  |  |
| pLYJ163 | Km^r^, vector for the expression of proteins in *E. coli* | Novagen |
| pTYQ01 | pLYJ163 plus *VBAly15A* | This study |
| pTYQ02 | pLYJ163 plus *VBAly15A^R114A^* | This study |
| pTYQ03 | pLYJ163 plus *VBAly15A^Y470A^* | This study |
| pTYQ04 | pLYJ163 plus *VBAly15A^R229A^* | This study |
| pTYQ05 | pLYJ163 plus *VBAly15A^R110A^* | This study |
| pTYQ06 | pLYJ163 plus *VBAly15A^H226A^* | This study |
| pTYQ07 | pLYJ163 plus *VBAly15A^Y280A^* | This study |
| pTYQ08 | pLYJ163 plus *VBAly15A^H446A^* | This study |
| pTYQ09 | pLYJ163 plus *VBAly15A^H226A/H446A^* | This study |
| pTYQ10 | pLYJ163 plus *VaAly17A^H174A^* | This study |
| pLY01 | pLYJ163 plus *VSAly15A* | Unpublished data |
| pLY02 | pLYJ163 plus *VSAly15B* | Unpublished data |

**Table S3 Primers used in this study**

| **Primers** | **Sequence (5'-3')** |
| --- | --- |
| **Construction of Expression Plasmid** | |
| *VBAly15A*-F | CTTTATTTTCAGGGCGCCATGGATGAGCAACGACAAAGCACT |
| *VBAly15A*-R | GTGGTGGTGGTGCTCGAGTTACAGTTCGATAGTAGCTG |
| *VBAly15A^R114A^*-F | TGGGCTCAAATGTACGTAGATTGC |
| *VBAly15A^R114A^*-R | TTGAGCCCAGTAAGGACGCCAAAG |
| *VBAly15A^Y470A^*-F | TGGTTACGCTGGTGGTTTCGGTGTAGACATGC |
| *VBAly15A ^Y470A^*-R | AACCACCAGCGTAACCAGTGATAGAAGCAAGAGTTTCA |
| *VBAly15A^R229A^*-F | CACGGTGTTGCTTCTATTTCTTCTGCTATTATCCCAACG |
| *VBAly15A^R229A^*-R | ATAGAAGCAACACCGTGGCTGTTAAGTGGGTT |
| *VBAly15A^R100A^*-F | TGGGCTCCTTACTGGCGTCAAATG |
| *VBAly15A^R110A^*-R | AGGAGCCCAAAGAGAAGCTTTACC |
| *VBAly15A^Y280A^*-F | GACGCTTGGAACACACAAACTGCA |
| *VBAly15A^Y280A^*-R | CCAAGCGTCAGGACCTTCAGCCCA |
| *VBAly15A^H226A^*-F | AGCGCTGGTGTTCGTTCTATTTCT |
| *VBAly15A^H226A^*-R | ACCAGCGCTGTTAAGTGGGTTGTT |
| *VBAly15A^H446A^*-F | AGCGCTTCTCACGCTGATCAAAAC |
| *VBAly15A^H446A^*-R | AGAAGCGCTGATAGAACCAAATGG |
| *VBAly15A^H226A/H446A^*-F | AGCGCTGGTGTTCGTTCTATTTCT |
| *VBAly15A^H226A/H446A^*-R | ACCAGCGCTGTTAAGTGGGTTGTT |
| *VaAly17A^H174A^*-F | TCACAACGCTGGCATCTGGGCAGTTGCGGCTG |
| *VaAly17A^H174A^*-R | AGATGCCAGCGTTGTGAATACGGTCGAAATCGT |

**References**

1. Ochiai A, Hashimoto W, Murata K. 2006. A biosystem for alginate metabolism in *Agrobacterium* *tumefaciens* strain C58: Molecular identification of Atu3025 as an exotype family PL-15 alginate lyase. *Res Microbiol* 157:642-649.

2. Lu D, Zhang Q, Wang S, Guan J, Jiao R, Han N, Han W, Li F. 2019. Biochemical characteristics and synergistic effect of two novel alginate lyases from *Photobacterium* sp. FC615. *Biotechnol Biofuels* 12:260.

3. Wataru Hashimoto 1 OM, Akihito Ochiai, Kousaku Murata. 2005. Molecular identification of *Sphingomonas* sp. A1 alginate lyase (A1-IV') as a member of novel polysaccharide lyase family 15 and implications in alginate lyase evolution. *J Biosci Bioeng* doi:10.1263/jbb.99.048.

4. Arora PK, Mori T, Takahashi M, Tanaka R, Miyake H, Shibata T, Chow S, Kuroda K, Ueda M, Takeyama H. 2016. *Falsirhodobacter* sp. alg1 harbors single homologs of endo and exo-type alginate Lyases efficient for alginate depolymerization. *PLoS One* 11.

5. Jagtap SS, Hehemann JH, Polz MF, Lee JK, Zhao H. 2014. Comparative biochemical characterization of three exolytic oligoalginate lyases from *Vibrio splendidus* reveals complementary substrate scope, temperature, and pH adaptations. *Appl Environ Microb* 80:4207-14.

6. Liu X-F, Cao Y, Zhang H-L, Chen Y-J, Hu C-J. 2015. Complete Genome Sequence of *Vibrio alginolyticus* ATCC 17749^T^. *Genome Announc* 3.

7. Zhang X, Tang Y, Gao F, Xu X, Chen G, Li Y, Wang L. 2024. Low-cost and efficient strategy for brown algal hydrolysis: Combination of alginate lyase and cellulase. *Bioresour Technol* 397.
